# Bilateral recurrent discloation of the patella associated with below knee amputation: A case report

**DOI:** 10.1186/1471-2474-6-9

**Published:** 2005-02-17

**Authors:** Sumit Batra, Ratnesh Kumar, Prasanna Lenka

**Affiliations:** 1 Department of Orthopaedics, National Institute for the Orthopaedically Handiccaped, B.T. Road, Bon Hooghly, Kolkata- 700090. India; 2 Director, National Institute for the Orthopaedically Handiccaped, B.T. Road, Bon Hooghly, Kolkata- 700090. India; 3 Department of Prosthetics & Orthotics, National Institute for the Orthopaedically Handiccaped, B.T. Road, Bon Hooghly, Kolkata- 700090. India

## Abstract

**Background:**

Recurrent dislocation of the patella in patients with below knee amputation is a known entity. Abnormally high-riding patella (patella alta) and medial patellofemoral ligament insufficiency in these patients predisposes them to patellar instability. The established treatment of this problem is surgical realignment.

**Case presentation:**

A 25 year old male patient with bilateral below knee amputation presented with bilateral recurrent dislocation of the patella while walking on knees on uneven ground. Clinical and radiographic studies showed patella alta. A simple shoe modification was used to treat this patient.

**Conclusions:**

A simple shoe modification can be used to treat such a condition which is otherwise treated surgically.

## Background

Recurrent dislocation of the patella can follow a violent initial dislocation, but occur more often in knees with one or more underlying anatomic abnormalities that predispose the patella to dislocation or subluxation. In these knees, less trauma is needed for dislocation to occur. The underlying pathologic condition causes an abnormal excursion of the extensor mechanism over the femoral condyles. High-riding patella (patella alta) and a damaged medial patellofemoral ligament at the time of first episode of dislocation leads to such an abnormality and leads to recurrent dislocation of the patella [1-4]. Patella alta has been reported in patients with below knee amputation using patellar tendon bearing prosthesis. The usual treatment in these cases is surgical reconstruction. We present a case of bilateral recurrent dislocation of the patella with below knee amputation which was managed conservatively.

## Case presentation

**Figure 1:**
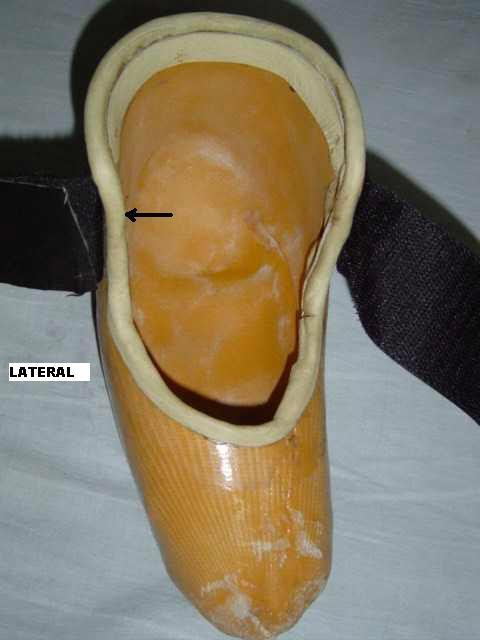
Close-up photograph of the shoe (arrow = moulding on the lateral side providing protective force against dislocation)

**Figure 2:**
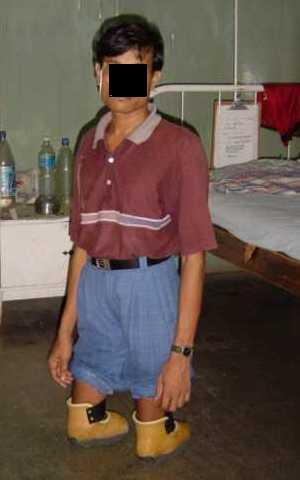
Patient wearing modified shoes

A 25 years old bilateral below knee amputee presented with recurrent dislocation of the patella while walking on knees in emergent situations on uneven ground without the prosthesis. Amputation was performed at the age of 15 years as a result of train accident. Since then he has been using patellar tendon bearing (PTB) type below knee prosthesis on both sides. First episode of dislocation occurred after 5 years of amputation. The patient used to walk on his knees without using the prosthesis for in-house activities on uneven ground. His patella used to dislocate whenever there was an unnoticed pressure on the medial side of the knee. On clinical examination, patella alta and positive apprehension test were noted on both sides. The ratio of patellar length to patellar tendon length was 0.8 on both sides demonstrating relative elongation of the patellar tendon. The normal ratio is 1.0 [1]. Modified shoes were given to the patient which were moulded in the inner surface around the patellar tendon and femoral condyles to provide uniform distribution of weight over a wider area. Another moulding was done on lateral side that prevented excessive movement of patella laterally [Fig. 1]. The patient was allowed to walk on knees after wearing these shoes [Fig. 2]. At 6 months follow up the patient is doing well with no recurrence.

## Discussion

High-riding patella (known as Patella alta) leads to patellar instability [1]. In the presence of such instability, sudden laterally directed forces can lead to dislocation of the patella. The first episode of lateral dislocation of patella invariably damages the medial patellofemoral ligament (MPFL) which is the primary passive restraint to lateral patellar displacement[4,5]. An injured MPFL leads to further instability and both the factors combined predispose to recurrent dislocation of the patella. In below knee amputees using patellar tendon bearing (PTB) prosthesis, the prolonged, upwardly directed force against the patellar tendon gradually elongate the tendon and produce patella alta [2,3]. It takes a long time for such an instability to develop. The first incidence of patellar dislocation occurred after 5 years of amputation in our patient. Similar time lag was described by Mowery et.al. in his patients where the time lag was from 5–13 years [3].

The patient described here used to walk on knees for certain emergent and short mobility in-house activities e.g., going to toilet especially at night, going to kitchen etc. As most of rural houses in developing countries do not have cemented floors, walking on uneven ground cannot be prevented. Walking on knees in such conditions produces eccentric forces on the patella. Sudden laterally directed forces which might result when the knee strikes some elevated surface on medial side can lead to dislocation of patella in patients with patellar instability.

Walking on knees is usually discouraged in below knee amputees as it can lead to flexion contractures of the knee joint. In cases of bilateral amputees it is not always possible for the patients to wear prosthesis on both sides in emergency situations as discussed above. Considering these points, the occasional walking on knees could not be avoided in this patient. Surgical correction of instability would not have helped much as walking on knees on uneven ground could have nullified the results of surgery. A modified well moulded shoe was given to the patient for walking on knees that prevented any eccentric forces and protected the patella against dislocation.

6 months follow-up results were excellent in this patient with no episode of dislocation on either side. The patient was very comfortable in using the shoes as it takes him only 15 seconds to wear them. Hence a simple low cost shoe modification has been used to treat a condition which is mostly treated surgically.

## Conclusions

Recurrent dislocation of the patella in cases of below knee amputees using patellar tendon bearing prosthesis is very rare and the usual treatment is surgical realignment. To our knowledge no case of bilateral recurrent dislocation of the patella in below knee amputees has been described in the literature. A simple shoe modification can be used in difficult situations to treat such a condition.

## Competing interests

The author(s) declare that they have no competing interests.

## Authors' contributions

SB was the principle surgeon who planned the treatment protocol of this patient, in addition to conceptualizing and drafting the article.

RK guided the designing of the shoe.

PL was the prosthetist who designed and made the shoe.

All authors read and approved the final manuscript.

## Pre-publication history

The pre-publication history for this paper can be accessed here:


